# The Comparative Effectiveness of Mobile Phone Interventions in Improving Health Outcomes: Meta-Analytic Review

**DOI:** 10.2196/11244

**Published:** 2019-04-03

**Authors:** Qinghua Yang, Stephanie K Van Stee

**Affiliations:** 1 Department of Communication Studies Bob Schieffer College of Communication Texas Christian University Fort Worth, TX United States; 2 Department of Communication and Media University of Missouri–St. Louis St. Louis, MO United States

**Keywords:** meta-analysis, mobile phones, mHealth, intervention study

## Abstract

**Background:**

As mobile technology continues expanding, researchers have been using mobile phones to conduct health interventions (mobile health—mHealth—interventions). The multiple features of mobile phones offer great opportunities to disseminate large-scale, cost-efficient, and tailored messages to participants. However, the interventions to date have shown mixed results, with a large variance of effect sizes (Cohen *d*=−0.62 to 1.65).

**Objective:**

The study aimed to generate cumulative knowledge that informs mHealth intervention research. The aims were twofold: (1) to calculate an overall effect magnitude for mHealth interventions compared with alternative interventions or conditions, and (2) to analyze potential moderators of mHealth interventions’ comparative efficacy.

**Methods:**

Comprehensive searches of the *Communication & Mass Media Complete*, *PsycINFO*, *Web of Knowledge*, *Academic Search Premier, PubMed* and *MEDLINE* databases were conducted to identify potentially eligible studies in peer-reviewed journals, conference proceedings, and dissertations and theses. Search queries were formulated using a combination of search terms: “intervention” (Title or Abstract) AND “health” (Title or Abstract) AND “*phone*” OR “black-berr*” (OR mHealth OR “application*” OR app* OR mobile OR cellular OR “short messag*” OR palm* OR iPhone* OR MP3* OR MP4* OR iPod*) (Title or Abstract). Cohen *d* was computed as the basic unit of analysis, and the variance-weighted analysis was implemented to compute the overall effect size under a random-effects model. Analysis of variance–like and meta-regression models were conducted to analyze categorical and continuous moderators, respectively.

**Results:**

The search resulted in 3424 potential studies, the abstracts (and full text, as necessary) of which were reviewed for relevance. Studies were screened in multiple stages using explicit inclusion and exclusion criteria, and citations were evaluated for inclusion of qualified studies. A total of 64 studies were included in the current meta-analysis. Results showed that mHealth interventions are relatively more effective than comparison interventions or conditions, with a small but significant overall weighted effect size (Cohen *d*=0.31). In addition, the effects of interventions are moderated by theoretical paradigm, 3 engagement types (ie, changing personal environment, reinforcement tracking, social presentation), mobile use type, intervention channel, and length of follow-up.

**Conclusions:**

To the best of our knowledge, this is the most comprehensive meta-analysis to date that examined the overall effectiveness of mHealth interventions across health topics and is the first study that statistically tested moderators. Our findings not only shed light on intervention design using mobile phones, but also provide new directions for research in health communication and promotion using new media. Future research scholarship is needed to examine the effectiveness of mHealth interventions across various health issues, especially those that have not yet been investigated (eg, substance use, sexual health), engaging participants using social features on mobile phones, and designing tailored mHealth interventions for diverse subpopulations to maximize effects.

## Introduction

### Background

As mobile technology continues to expand and mobile phones become ubiquitous, with 73% of Americans actively using mobile phones [1] and approximately 7 billion mobile phone subscriptions worldwide [2], mobile phones are being increasingly used to conduct health interventions (mobile health—mHealth—interventions) to improve health conditions [3]. Studies have found several advantages of mHealth interventions compared with traditional approaches. Given the number of mobile phone users, mHealth interventions have the potential to engage a large group of people at a relatively lower cost, making public health interventions more feasible and impactful [3-5]. As many mHealth intervention platforms (eg, text-based, apps) have become available to benefit interventions in differing ways [1], mHealth can now address many issues including a limited workforce, finances, and accessing difficult-to-reach groups [6]. Overall, the use of mobile phones as a part of health interventions has become an effective tool to potentially prevent and treat health issues [6].

Despite the promises of mHealth interventions, previous mHealth interventions have yielded conflicting results and inconsistent effect sizes (ESs), ranging from Cohen *d* of −0.62 [7] to 1.65 [8]. Such large variance in ESs makes a comprehensive meta-analysis with moderator analyses imperative to provide a clearer picture of the effectiveness of mHealth interventions. However, previous syntheses either focused on a specific health issue [9,10] or were constrained by small sample sizes and a lack of moderator analyses [3,4], leaving what factors account for variance in ESs unanswered.

To fill the gap and provide insights into the effectiveness of harnessing mobile technology for health interventions, we aim to do the following: (1) calculate an overall effect magnitude for mHealth interventions compared with alternative interventions or conditions, and (2) analyze potential theoretical, methodological, and demographic moderators of mHealth interventions’ comparative effectiveness. To achieve these goals, meta-analysis was implemented as the research method, which enables researchers to conduct a sophisticated synthesis of quantitative research literature [11]. Applying this innovative approach, we identify the overall effect of mHealth interventions and significant moderators to provide guidance for mHealth intervention design and implementation.

### Mobile Phone Use and Mobile Health

With the growing technological culture, mobile phones have become the most popular and widespread personal technology, with 95% of the Americans owning a cell phone of some kind and 77% owning a smartphone [12]. Mobile technologies also include personal digital assistant phones (eg, BlackBerry), portable media players (eg, MP3- and MP4-players), and handheld computers (eg, iPad). As the use of mobile technology has increased, public health professionals have begun to take advantage of the multiple platforms provided by mobile phones to serve a wide variety of purposes, such as physical activity, weight loss, smoking cessation, mental health, and chronic disease management.

“The use of mobile computing and communication technologies in health care and public health” [10] is referred to as mHealth, a rapidly expanding branch within eHealth. There are many effective strategies that utilize mobile phones to promote public health. In general, mobile phones have been used to share information about public health as it is economical, sustainable, and effective [13]. To benefit mobile phone users, public health professionals have begun to utilize mobile phone capabilities for prevention, management, and treatment of health issues [14]. Mobile phones have been primarily applied for health purposes through short message service (SMS) and app features of these technological devices. In particular, text messages sent through mobile phones were found as a simple and efficient option for health services, which could be affordably used to send tailored health messages and reminders to improve delivery to patients [15-17]. Mobile apps are also widely used to promote public health, which can integrate a variety of built-in interactive features. Therefore, this allows for the potential to target heterogeneous audiences to address specific needs with diverse outcomes [15]. These apps offer great potential for dynamic engagement of patients and providers in health care and an innovative approach to improve health outcomes [18].

### Mobile Health Interventions

Health interventions have utilized mobile technology in a variety of ways to increase health knowledge, promote health education, and change health behaviors. With the increasing use of mHealth interventions, several approaches have emerged for utilizing mobile technologies to implement health interventions. A primary approach to incorporating mobile phones into health interventions is focusing on the voice and text features that have achieved significant improvements in compliance with medication adherence, asthma symptoms, glycated hemoglobin (HbA_1c_) levels, stress levels, smoking cessation, and self-efficacy [19]. Leveraging the advantages of mHealth interventions has improved public health outcomes in these areas as it has helped individuals become more aware and take accountability for their health issues. Other approaches to mHealth interventions include self-monitoring techniques and real-time surveillance features of the technology [20]. It is increasingly popular to utilize mobile phones to track individuals’ daily activity and provide reminders and motivational text-based messages to continue progression during the intervention [21]. As smartphones have become more popular, another approach is the social networking component that allows users to interact and share information using social media [22]. With the booming marketplace and the engaging features of mobile apps, mHealth interventions have been increasingly based on mobile apps to deliver health information and modify health behavior and have significant influence on youths’ health outcomes [1].

Mobile phones have become a source of interactive communication, providing numerous advantages in conducting health interventions, including widespread use of mobile technology across various socioeconomic groups, few geographical constraints compared with other media, and cost-effectiveness to reach a diverse and large population [2,23]. A unique and noteworthy advantage of mHealth interventions is the ability to target underdeveloped or underserved areas due to the enabling resources provided by mobile phones [14,24]. A review of mobile phone-based health interventions for noncommunicable disease management in sub-Saharan Africa reported that using apps on cellular phones can improve physical and mental health outcomes [14]. Incorporating mobile phones within public health education and promotion can be beneficial for a wide range of situations and populations.

### Mixed Effects of Mobile Health Interventions and Research Questions

Despite mHealth interventions’ great potential to be superior to health interventions using traditional approaches, empirical research to date has generated mixed results in terms of the efficacy of mHealth interventions. For example, one successful mHealth intervention led to lower levels of perceived stress for intervention participants relative to a waitlist control group (Cohen *d*=1.02) at 6-month follow-up [25]. However, other mHealth interventions performed no better or worse than comparison conditions. For example, Cho et al [26] found that an mHealth intervention was less successful than a control condition (Cohen *d*=−0.24) at improving fasting plasma glucose levels of patients with type 2 diabetes. Such inconsistent findings make the efficacy of mHealth interventions in improving health outcomes unclear and indicate the necessity for a meta-analytic review.

To assess the overall comparative effect of mHealth interventions, the first research question (*RQ1*) was posed as follows: What is the overall effect magnitude for mHealth interventions compared with alternative interventions or conditions in improving health outcomes?

To take a close examination of the large variation of ESs in the efficacy of mHealth interventions, the first potential moderating variable is the theoretical framework applied in these studies. Theory enriches and provides a roadmap for research practices [5]. Therefore, it is plausible to assume that theory-based mHealth interventions may be more efficacious than their counterparts. A wide variety of theories have been applied in the mHealth interventions to date, including behavioral theories (eg, health belief model, HBM [27]; theory of planned behavior, TPB [28]), cognitive theories (eg, social cognitive theory, SCT [29]), or behavioral and cognitive theories (eg, cognitive behavioral therapy, CBT [30]). However, which theoretical paradigm works best in mHealth interventions remains unclear, especially when previous meta-analyses documented that the use of theory to guide intervention development does not significantly moderate ESs [1].

Besides investigating the theoretical framework, previous research [31-33] suggests that health topic, intervention designs (eg, control group design, length of intervention and follow-up), and participants’ features (eg, age, gender, and health conditions) could moderate the effects of health interventions. Specifically, a meta-analysis on health interventions using social networking sites reported that studies using a *true* control condition without giving any intervention had a significantly higher weighted mean ES than studies giving an alternative intervention to the control group [33]. To examine the comparative effectiveness instead of the absolute effectiveness of mHealth interventions, it is crucial to take into consideration the control group design, including regular treatment [34], print-version [35] or computer-version [36] interventions, less intensive version of mHealth interventions [37], or interventions combining multiple channels [38]. Therefore, these methodological and demographic variables suggested by previous studies will be analyzed as potential moderators.

In addition, several mobile-phone-related features will also be analyzed as potential moderators. First, mobile phones have been applied in health interventions through different strategies. Some studies only used SMS [38,39] or mobile apps [40,41], whereas others combined both SMS and mobile apps [42,43]. Second and relatedly, there are not only interventions that applied mobile phone as the only channel [7,17] but also those that combined mobile phone with either face-to-face communication [39,44], another type of media [8,37], or both [34,45]. Although some researchers suggested that unimodal interventions could provide participants with more exposure and be easier to manage [46,47], leading to higher effectiveness, others advocated for multimodal interventions, which are more likely to engage participants and therefore function better in health promotion [48] or reported no difference between them [1]. Furthermore, Sama et al proposed a typology of 8 types of mobile phone engagement—changing personal environment, facilitating social support, goal setting, progress tracking, reinforcement tracking, self-monitoring, social presentation, and social referencing [18]. However, the types of engagement that improve the effectiveness of mHealth interventions remain unclear and will be examined in this study.

*RQ2*: Is the comparative effectiveness of mHealth interventions moderated by (1) theoretical paradigm, (2) health topics, (3) types of engagement, (4) mobile use type, (5) intervention channel, (6) control group design, (7) length of intervention, (8) length of follow up, and (9) participants’ features?

## Methods

### Literature Search

**Figure 1:**
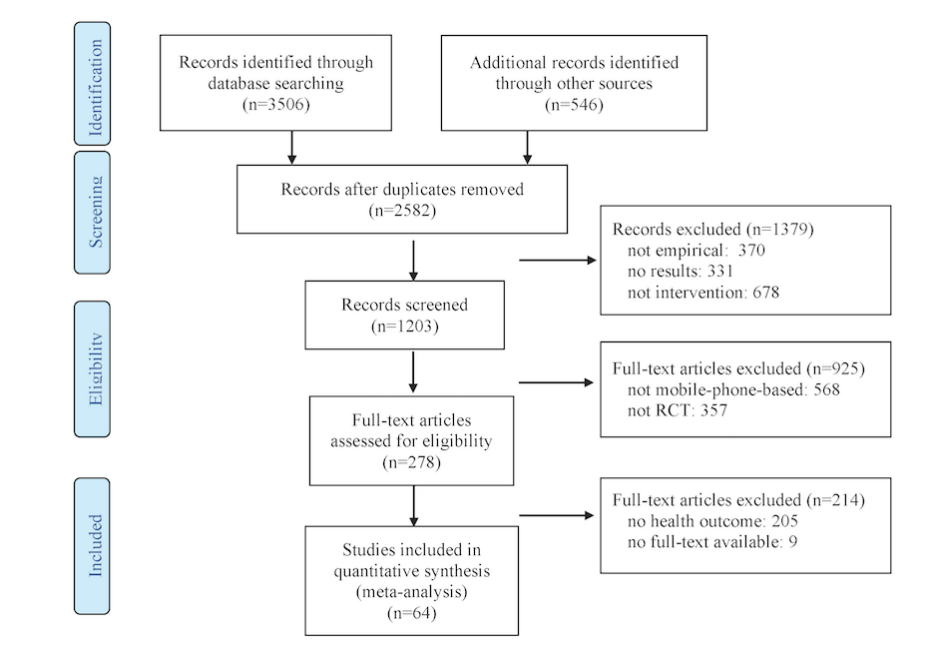
Summary of selection process used in this study. Interventions using mobile phones only for data collection or making phone calls were excluded in this meta-analysis. RCT: randomized controlled trial.

To provide a clear picture of mHealth interventions’ effectiveness in improving health outcomes, comprehensive searches of the *Communication & Mass Media Complete*, *PsycINFO*, *Web of Knowledge*, *Academic Search Premier, PubMed* and *MEDLINE* databases were conducted to identify potentially eligible studies in peer-reviewed journals and conference proceedings as well as dissertations and theses, which have been published through December 31, 2017. Search queries were formulated using a combination of search terms: “intervention” (Title or Abstract) AND “health” (Title or Abstract) AND “*phone*” OR “black-berr*” (OR mHealth OR “application*” OR app* OR mobile OR cellular OR “short messag*” OR palm* OR iPhone* OR MP3* OR MP4* OR iPod*) (Title or Abstract). We retrieved 3506 studies from the databases, the abstracts (and full text, as necessary) of which were reviewed for relevance. Studies were screened in multiple stages using explicit inclusion and exclusion criteria (see Figure 1). We also screened the primary research articles included in a systematic review of mHealth-focused systematic reviews (n=546; [49]) to evaluate them for inclusion.

### Overview of Meta-Analysis

As generally recommended in the meta-analysis methodological literature [50,51], Cohen *d* was computed as the basic unit of analysis for the meta-analytic review. The statistical analyses were based on methods proposed by Hedges and Olkin [52]. As publication bias may exist when the publication status depends on the statistical significance of study results [53], multiple analytic approaches were implemented to check for publication bias. First, a funnel plot was used to examine whether ESs from smaller studies show more variability than those from larger studies. Given that the funnel plot interpretation was open to subjectivity, Rosenthal’s Fail-safe *N* and Duval and Tweedie’s Trim and Fill method were also applied to provide statistical evidence of publication bias.

The current meta-analysis used the variance-weighted analysis [52]: the overall weighted ES was computed by weighting the unbiased ES (*d*) by the inverse of its associated variance (Wi=1/Vi). The overall homogeneity of ESs was tested using *Q* statistics to determine whether all effects were from the same population. When *Q* statistics are significant, the ESs are not from the same population, and the overall ES should be computed under the random-effects models (REMs), which incorporates between-studies uncertainty in the computation [54]. Otherwise, fixed-effects model (FEM) would be used.

In the moderator analysis, analysis of variance–like categorical models were conducted to analyze categorical moderators (eg, health topic, mobile use type) using mixed-effects models (MEM), as FEM with categorical moderator assumes that all studies in 1 subgroup share a common ES, whereas the MEM allows true variation of effects within subgroups of studies [55]. The same approach was applied when using meta-regression modeling to analyze continuous moderators (eg, length of follow-up and participants’ age). In the cases where moderator analyses were statistically significant under the MEM, posthoc analysis was conducted for pairwise comparison using Tukey contrasts with adjusted *P* value. The analyses were conducted using *Metafor* and *Multcomp* package in R software (R Foundation for Statistical Computing).

## Results

### Study Description

A total of 64 studies were included in the current meta-analysis (see Multimedia Appendix 1), with 142 ESs computed following Schmidt and Hunter's approach [56]. Among the 64 studies, 47 were based on at least one theory, including SCT [7,8,26,30,35,37,39,40,57-73], transtheoretical model [17,38,58,74-78], self-regulation theory [39,68,79], self-determination theory [42,43], HBM [45,57,66,80], and theory of reasoned action and/or TPB [7,8,40,60]. The meta-analyzed mHealth interventions focused on 5 topics, namely mental health [25,36,45,48,56,81-84], nutrition and weight status [17,34,35,38,39,42,57,69,74-76], physical activity [7,8,26,37,56,57,74-76,78,79,84,85], health-related quality of life and well-being [47,61,73,86], and chronic disease management [8,23,26,40,44,58,62,63,72,75,77,79,87-99]. The categorization of health topics was based on Healthy People 2020 [100]. There were originally 8 categories in the code book (ie, 1=tobacco use, 2=mental health, 3=a nutrition and weight status, 4=physical activity, 5=sexual health, 6=health-related quality of life and well-being, 7=HIV/AIDS, 8=chronic disease management). However, as no study focused on tobacco use, sexual health, or HIV/AIDS, these 3 categories were excluded from analyses. There were 10,296 (*N*=10,296) participants across included studies.

### Publication Bias

**Figure 2:**
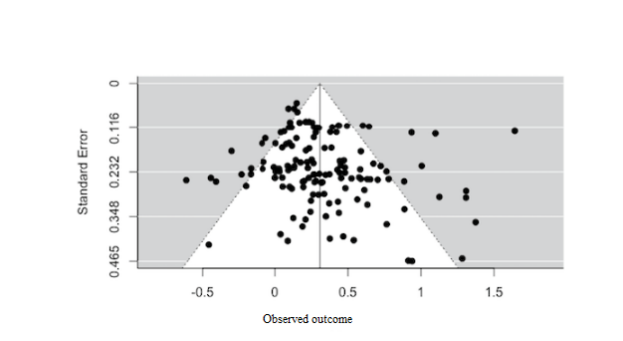
Funnel plot of effect sizes to check publication bias for this study.

Publication bias may exist when publication status depends on the statistical significance of study results [52]. We have applied multiple techniques to check for potential publication bias. First, a funnel plot can be used to examine whether ESs from smaller studies show more variability than those from larger studies. As shown in Figure 2, the funnel plot of ESs seems to be generally symmetric, which is consistent with the Regression Test for funnel plot asymmetry (*z*=1.51, *P*=.13) and provides evidence for the absence of publication bias. Rosenthal’s Fail-safe *N* was 17,539, which is much larger than the tolerance level (5*k* +10=660), and no study was found missing for symmetry using Duval and Tweedie’s Trim and Fill Method, which further confirmed the absence of publication bias.

### Overall Analysis

Estimated under the FEM, the *Q* statistic was significant (*Q*_*total*_ (*df*=141)=467.01, *P*<.001), indicating that the ESs were not homogeneous, and mean ES was estimated under the REM using Restricted Maximum Likelihood Estimation method. Under the REM, the sample weighted mean for standardized mean difference was 0.31 (95% CIs; 0.25, 0.36), which is a small ES [101], but statistically significant (*P*<.001). In other words, there was a statistically significant mean difference between the mHealth intervention and control groups according to the overall analysis. *I*^*2*^, an index representing the ratio of true heterogeneity to total variance across observed ESs, is 71.87%, indicating large between-study variance [102]. Similarly, Birge ratio, another index to quantify the magnitude of heterogeneity (computed as *Q* /*df*=467.01/141=3.31), is larger than 1 (the ratio when all the variance comes from sampling error), indicating large between-study heterogeneity. Sampling error variance (*S*_*e*_^2^=.0135) only accounted for 18.91% of the total variance (*S*^2^=.0714), suggesting the presence of a moderator. Therefore, the moderators proposed in *RQ2* were analyzed.

### Moderator Analyses

Moderator analyses were conducted by analyzing *theoretical paradigm* (1=no theory, 2=behavioral theory, 3=cognitive theory, 4=behavioral and cognitive theories combined), *health topic* (1=mental health, 2=nutrition and weight status, 3=physical activity, 4=health-related quality of life and well-being, 5=chronic disease management), *eight types of engagement* [18], *mobile use type* (1=text messages, 2=mobile app, 3=combined), *intervention channel* (1=mobile phone only, 2=mobile phone combined with other type of media, 3=mobile phone combined with face-to-face communication, 4=mobile phone combined with other type of media and face-to-face communication), *control group design* (1=no intervention, eg, waiting list group; 2=intervention based on interpersonal communication [no media involved, eg, counseling at clinic], 3=intervention using other type of media than mobile phone, eg, website; 4=intervention using mobile phone, eg, text messages about general health information instead of targeted health behavior; 5=intervention with multiple features), and *participants’ health condition*: (1=general healthy adults, 2=population at risk) as categorical moderators respectively. Moreover, *participants’ mean age*, *percentage of female participants* (to examine the influence of gender), *length of the intervention*, and *length of follow-up* were analyzed as continuous moderators.

#### Theoretical Paradigm

Under MEM, theoretical paradigm was significant as a moderator (*Q*_*between*_ (*df*=3)=11.01, *P*=.01). Posthoc pairwise comparison indicated that mHealth interventions based on cognitive and behavioral theories combined (*d*=0.45, *P*<.001) had the highest weighted mean ES among the 4 categories and was significantly higher than interventions not indicating a theory (*d*=0.28, *P*<.001) or interventions applying behavioral (*d*=0.23, *P*<.001) both at .05 level or cognitive theory only (*d*=0.20, *P*<.001) at .01 level.

#### Health Topic

Under MEM, health topic was not a significant moderator (*Q*_*between*_ (*df=* 4)=1.63, *P*=.80), with mHealth interventions on physical activity showing the lowest weighted mean ES (*d*=0.24; SE=0.06; 95% CIs 0.11, 0.36; *P*<.001) and interventions on nutrition and weight status showing the highest ES (*d*=0.36; SE=.08; 95% CIs 0.21, 0.52; *P*<.001), which however are not significantly different from each other. The weighted mean ESs across all 5 topics were significantly larger than zero.

#### Types of Engagement

Among the 8 types of engagement proposed by Sama et al [18], only changing personal environment (*Q*_*between*_ (*df*=1)=9.44, *P*=.002), reinforcement tracking (*Q*_*between*_ (*df*=1)=10.24, *P*=.001), and social presentation or announcement (*Q*_*between*_ (*df*=1)=6.42, *P*=.01) were significant moderators. Specifically, the weighted mean ES of the mHealth interventions with the function of changing personal environment (*d*=0.76; SE=0.15; 95% CIs 0.47,1.05; *P*<.001) was significantly higher than that of the interventions without this feature (*d=* 0.29; SE=0.03; 95% CIs 0.24, 0.35; *P*<.001) at .01 level (*z*=3.11). Similarly, the weighted mean ES of the mHealth interventions with the reinforcement tracking function (*d*=0.43; SE=0.05; 95% CIs 0.33, 0.53; *P*<.001) was significantly higher than that of the interventions without this function (*d*=0.24; SE=0.03; 95% CIs 0.18, 0.30; *P*<.001) at .01 level (*z*=3.18). However, the weighted mean ES of the mHealth interventions with the social presentation or announcement function (*d*=0.04; SE=0.08; 95% CIs -0.13, 0.20; *P*=.65) was significantly lower than that of the interventions without (*d*=0.33; SE=.03; 95% CIs 0.27, 0.38; *P*<.001) at .05 level (*z*=2.53).

#### Mobile Use Type

How the mobile phone was applied in the intervention was found as a significant moderator (*Q*_*between*_ (*df*=2)=17.35, *P*<.001). Post hoc analysis indicated that the weighted mean ES of interventions combining SMS and mobile apps (*d*=0.59; SE=.12; 95% CIs 0.36, 0.83; *P*<.001) was significantly higher than the ESs of those using only SMS (*d*=0.30; SE=0.04; 95% CIs 0.23, 0.38; *P*<.001) or mobile apps (*d*=0.22; SE=.03; 95% CIs 0.16, 0.29; *P*<.001; *Z*_*SMS*_=3.37, *Z*_*App*_=4.16).

#### Intervention Channel

The channel through which mHealth interventions were implemented turned out to be another significant moderator (*Q*_*between*_ (*df*=3)=12.56, *P*=.006). Pairwise comparison indicated that the weighted mean ES of interventions combining mobile phone with another type of media (*d*=0.39; SE=.05 95% CIs 0.30, 0.49; *P*<.001) was significantly higher than that of interventions using mobile phone only (*d*=0.19; SE=.04; 95% CIs 0.12, 0.26; *P*<.001; *z*=2.63) or interventions combining mobile phone with face-to-face communication (*d*=0.18; SE=.04; *K*=14; 95% CIs 0.10, 0.25; *P*<.001; *z*=2.74).

#### Control Group Design

Nonsignificant differences between-study variance under MEM (*Q*_*between*_ (*df*=4)=5.21, *P*=.27) indicated that control group design did not show a significant difference in the comparative effectiveness of the interventions.

#### Participants’ Age, Gender, and Health Condition

The majority of mHealth interventions in the current sample were conducted with at-risk populations, except for 6 studies [7,42,71,61,78,85]. However, whether the participants were healthy or at-risk populations was not a significant moderator of the ESs (*Q*_*between*_ (*df*=1)=2.31, *P*=.13). Neither participants’ age (*Q*_*between*_ (*df=* 1)=.99, *P*=.32) nor gender (*Q*_*between*_ (*df=* 1)=1.94, *P*=.16) was a significant moderator.

#### Length of Intervention and Follow-Up

Intervention length was a nonsignificant moderator (*Q*_*between*_ (*df*=1)=0.57, *P*=.45); however, when the follow-up measures were conducted, it did moderate the ESs (*Q*_*between*_ (*df*=1)=13.46, *P*<.001). Length of follow-up ranged from immediate [57] to 9 months later [71], with an average follow-up period being 2.48 weeks (SD=6.31). The weighted mean ES was significant immediately after the intervention (*d*=0.27, *P<*.001), and increased by .015 for each additional week of follow-up (*P*<.001).

## Discussion

### Overall Effects

Findings from this meta-analysis indicated that mHealth interventions are significantly more effective than comparison conditions at improving health outcomes (*d*=0.31; 95% CIs 0.25, 0. 36), which is consistent with previous meta-analyses focusing on specific health issues [9,103,104]. In particular, mHealth interventions have been significantly more effective than comparison conditions for physical activity (Hedge *g*=0.54; 95% CIs 0.17, 0.91] [9] and led to significant improvement in diabetes management (mean 0.5% reduction in HbA_1c_) [104]. As it relates to SMS, text interventions were more effective for antiretroviral therapy adherence than control conditions (OR 1.39, CI 1.18, 1.64) [103]. Our finding related to the relative effectiveness of mHealth interventions shows not only consistency with previous research but extends current research by examining the effects across health contexts. In addition to the significant overall effect of mHealth interventions, several moderators were identified, which help explain the mechanisms behind the variance in mHealth interventions’ efficacy.

### Effects of Engagement

Findings indicated several statistically significant moderators of mHealth intervention effects. Of these, 1 such moderator is the type of engagement (ie, changing personal environment, reinforcement tracking, and social presentation or announcement). mHealth interventions that included features for changing one’s personal environment and/or reinforcement tracking exhibited larger relative effects than mHealth interventions without those features. Changing a person’s personal environment directly enables people to engage in the desired behavior or a behavior that affects the desired health outcome (eg, soothing sounds for meditating, which may help with stress) [18]. Resources that allow users to immediately engage in the desired behavior (or a behavior that affects the desired health outcome) may help to reduce barriers that may otherwise prevent them from engaging in healthy behaviors that improve health outcomes. Theories of health behavior change (eg, HBM and TPB) address the important role that perceived or actual barriers play, indicating that reducing perceived or actual barriers can enhance the likelihood of positive behavior change. Alternatively, mHealth interventions that included the feature of social presentation or announcement exhibited smaller relative effects than mHealth interventions without that feature. This finding is counterintuitive, as one might expect that a social presentation or announcement feature would have greater positive effects on health outcomes, as providing information to others about one’s accomplishments may increase motivation [105]. A possible explanation for this is that social presentation or announcement may serve as a distraction to participants. Previous research has found that features intended to draw in or engage audiences may actually distract them from the position advocated by messages [106]. Alternatively, the lower relative effects of mHealth interventions with a social presentation or announcement feature may be due largely to the 1 study with this feature that contributed the largest negative ES in the meta-analysis [7].

### Effects of Intervention Channel

mHealth interventions using both SMS and a mobile app were relatively more effective than interventions using either SMS or a mobile app. SMS were typically used to collect data about participants’ behavior [45], and/or provide reinforcement for desired behaviors [67,85]. Furthermore, mHealth interventions that included other media were relatively more effective than interventions that included face-to-face components. Many of the mHealth interventions that included additional media channels used websites, emails, and/or print materials. Interventions with face-to-face communication as an additional channel typically used in-person health care or counseling or group workshops. mHealth interventions incorporating other media may be more effective because they provide additional content exposure, use complementary strategies, and/or drive people to the mobile intervention components, which is helpful in delivering messages to users with a variety of media use habits [107], especially for those whose preferred medium is not mobile phone.

### Length of Follow-Up

mHealth interventions with a longer follow-up exhibited greater effects than those with a shorter follow-up. Previous research indicates that length of follow-up can serve as a moderator of relative intervention effects [31]; however, in previous research, greater effectiveness was shown for shorter-term follow up [33], which is the opposite of the findings of this study. The finding may reflect the fact that some mHealth intervention studies used apps that are freely available, commercial apps [68], which participants could continue using after the intervention period. This finding highlights the promise of mHealth interventions in promoting positive long-term health outcomes. The easy accessibility and cost efficiency of mobile features may help prevent diminishing intervention effects compared with those that use other types of new media [33].

### Theoretical and Practical Implications

Our moderator analyses found that studies based on both cognitive and behavioral theories were more effective than those based on no theory or behavioral or cognitive theory only. Cognitive theories, such as SCT [29] or self-regulation theory [108], mainly focus on psychological factors and inner thoughts. Although they are related to behavior, there is still a gap between intention and actual behavior due to internal and external barriers [28], and how to bridge this gap and eventually trigger behavioral change are not well-addressed. Given this, scholars have critiqued the use of traditional psychosocial theories, which could fail to maximize the opportunities offered by mHealth interventions and called for closer assessment of each mHealth program and user engagement [109]. On the other hand, perceived and actual barriers are key variables in behavioral theories, such as HBM [27] or TPB [28], which acknowledge and emphasize reducing these barriers to achieve positive behavioral change. However, behavior is rooted in cognitions, which are considered precursors to behavioral change; positive behavioral change that is lacking a strong cognitive foundation may not have longitudinal effects. Therefore, the complementarity of cognitive and behavioral theories may explain the large ESs of studies that applied both types of theories [62,93]. The convergence of cognitive and behavioral theories has observed success in CBT [110] and deserves further investigation by health communication researchers.

In terms of mHealth intervention design, given that tailored communication is effective in promoting health behavior change and health outcomes [31], it is important to enable users to change their personal environment using mobile phones and provide personalized reinforcement messages based on users’ progress on health outcomes. According to the Elaboration Likelihood Model [111], personally relevant messages are more likely to increase personal involvement and trigger central route message processing, which would achieve stable persuasive effects over time. Alternatively, health researchers and professionals should take caution when incorporating social presentation or announcement features to engage participants and to avoid distraction. More research examining how to strategically integrate social engagement features [18] into mobile phones without affecting message exposure [46,47] to improve health outcomes is needed.

When designing mobile interventions, it is worth considering combining both SMS and app features, which are available on most mobile phones. SMS is easier to implement, whereas apps could afford multimedia interactive features; such features would complement each other to potentially maximize user engagement and health outcomes. According to channel complementary theory [103], people use multiple sources, which serve different niches and present unique information, to acquire information in certain health topics. Therefore, health researchers and professionals could also take advantage of other types of media when designing mHealth interventions, especially internet, through which several studies have achieved high efficacy [8,25,36]. Finally, due to the easy accessibility and cost efficiency of mobile phones, our finding also highlights the promise of mHealth interventions in achieving long-term health effects. Thus, researchers aiming to improve health outcomes over a long period could base interventions on existing low-cost mobile apps or free SMS to enable sustained use of the target mobile features and consequently maintain positive effects.

### Limitations and Future Research

Despite this study’s pioneering efforts, several limitations should be noted. First, although this study started from a comprehensive literature search and included more studies than previous reviews ([3,4,10], the sample size of several specific categories remains small. For instance, none of the studies meeting inclusion criteria focused on reducing substance use, promoting sexual health, or preventing HIV/AIDS. Among the health issues in the current meta-analysis, only 4 studies focused on health-related quality of life [47,61,72,112]. In the same vein, both the social presentation [7,48,61,68] and social referencing [7,8,48,85] engagement types have been applied in only 4 studies. The comparatively small sample sizes of the randomized controlled trials (RCTs)—attributed to the recency of mHealth interventions and the long implementing and publishing process—could limit the reliability of statistical results in specific categories and increase the likelihood of chance differences. As such, more empirical evidence is needed to have a more reliable estimation of the moderating effects in mHealth interventions, especially in understudied areas.

Second, in an attempt to be as comprehensive as possible, this study included not only published studies but also conference papers and unpublished work, which makes the study vulnerable to inclusion of lower quality studies, a general limitation for meta-analytic research [54]. However, only 2 included studies contributing 5 ESs were dissertations; all other included studies appeared in peer-reviewed journal articles.

Finally, despite efforts to include only the most relevant RCT studies, this study is susceptible to the “apples and oranges” critique concerning the comparability of studies, a common concern for meta-analytic reviews [54]. Comparability refers to all the studies included in one meta-analysis examining the same constructs or relationships. Due to the relatively small sample size in some categories, such as the variety of specific media that were used in combination with mobile phones in interventions, they were grouped in a larger category for moderator analyses.

### Conclusions

mHealth interventions have become increasingly common in recent years, given the ubiquity of mobile phone ownership, providing the possibility to tailor health messages, cost efficiency of mobile delivery channels, and opportunity for large-scale dissemination. To the best of our knowledge, this is the most comprehensive meta-analysis to date that examined the overall effectiveness of mobile phone interventions across health topics and is the first study across health behaviors that statistically tested moderators. By analyzing 64 studies, we found that mHealth interventions have a small but significant weighted mean ES (Cohen *d*=0.31), which is moderated by theoretical paradigm, engagement types (ie, changing personal environment, reinforcement tracking, and social presentation), mobile use type, intervention channel, and length of follow-up. Our findings not only shed light on intervention design using mobile phones, but also provide new directions for research in health communication and promotion using new media. Future research is needed to examine the effectiveness of mHealth interventions across various health issues, especially those that have not yet been fully investigated (eg, substance abuse and sexual health), engaging participants using social features on mobile phones, and designing tailored mHealth interventions for diverse subpopulations to maximize effects.

## Appendix

Multimedia Appendix 1 Summary of studies included in the meta-analysis.

## Abbreviations

CBT: cognitive behavioral therapy
ES: effect size
FEM: fixed-effects model
HbA _1c_: glycated hemoglobin
HBM: health belief model
MEM: mixed-effects model
mHealth: mobile health
RCT: randomized controlled trial
REM: random-effects model
RQ: research question
SCT: social cognitive theory
SMS: short message service
TPB: theory of planned behavior

## References

[1] Fedele DA, Cushing CC, Fritz A, Amaro CM, Ortega A (2017). Mobile health interventions for improving health outcomes in youth: a meta-analysis. JAMA Pediatr.

[2] Voth EC, Oelke ND, Jung ME (2016). A theory-based exercise app to enhance exercise adherence: a pilot study. JMIR Mhealth Uhealth.

[3] Hall AK, Cole-Lewis H, Bernhardt JM (2015). Mobile text messaging for health: a systematic review of reviews. Annu Rev Public Health.

[4] Free C, Phillips G, Galli L, Watson L, Felix L, Edwards P, Patel V, Haines A (2013). The effectiveness of mobile-health technology-based health behaviour change or disease management interventions for health care consumers: a systematic review. PLoS Med.

[5] Glanz K, Rimer B, Viswanath K (2008). Health Behavior And Health Education: Theory, Research, And Practice.

[6] Beratarrechea A, Lee AG, Willner JM, Jahangir E, Ciapponi A, Rubinstein A (2014). The impact of mobile health interventions on chronic disease outcomes in developing countries: a systematic review. Telemed J E Health.

[7] Du H, Venkatakrishnan A, Youngblood GM, Ram A, Pirolli P (2016). A group-based mobile application to increase adherence in exercise and nutrition programs: a factorial design feasibility study. JMIR Mhealth Uhealth.

[8] Block G, Azar KM, Romanelli RJ, Block TJ, Hopkins D, Carpenter HA, Dolginsky MS, Hudes ML, Palaniappan LP, Block CH (2015). Diabetes prevention and weight loss with a fully automated behavioral intervention by email, web, and mobile phone: a randomized controlled trial among persons with prediabetes. J Med Internet Res.

[9] Fanning J, Mullen SP, McAuley E (2012). Increasing physical activity with mobile devices: a meta-analysis. J Med Internet Res.

[10] Free C, Phillips G, Watson L, Galli L, Felix L, Edwards P, Patel V, Haines A (2013). The effectiveness of mobile-health technologies to improve health care service delivery processes: a systematic review and meta-analysis. PLoS Med.

[11] Noar SM (2006). In pursuit of cumulative knowledge in health communication: the role of meta-analysis. Health Commun.

[12] (2018). Pew Research Center.

[13] Brinkel J, Krämer A, Krumkamp R, May J, Fobil J (2014). Mobile phone-based mHealth approaches for public health surveillance in Sub- Saharan Africa: a systematic review. IJERPH.

[14] Opoku D, Stephani V, Quentin W (2017). A realist review of mobile phone-based health interventions for non-communicable disease management in sub-Saharan Africa. BMC Med.

[15] Fiordelli M, Diviani N, Schulz PJ (2013). Mapping mHealth research: a decade of evolution. J Med Internet Res.

[16] Montes JM, Medina E, Gomez-Beneyto M, Maurino J (2012). A short message service (SMS)-based strategy for enhancing adherence to antipsychotic medication in schizophrenia. Psychiatry Res.

[17] Shaw R (2012). Duke University.

[18] Sama PR, Eapen ZJ, Weinfurt KP, Shah BR, Schulman KA (2014). An evaluation of mobile health application tools. JMIR Mhealth Uhealth.

[19] Krishna S, Boren SA, Balas EA (2009). Healthcare via cell phones: a systematic review. Telemedicine and e-Health.

[20] Tate EB, Spruijt-Metz D, O'Reilly G, Jordan-Marsh M, Gotsis M, Pentz MA, Dunton GF (2013). mHealth approaches to child obesity prevention: successes, unique challenges, and next directions. Transl Behav Med.

[21] Martin SS, Feldman DI, Blumenthal RS, Jones SR, Post WS, McKibben RA, Michos ED, Ndumele CE, Ratchford EV, Coresh J, Blaha MJ (2015). mActive: a randomized clinical trial of an automated mHealth intervention for physical activity promotion. J Am Heart Assoc.

[22] Maher C, Ferguson M, Vandelanotte C, Plotnikoff R, De Bourdeaudhuij I, Thomas S, Nelson-Field K, Olds T (2015). A web-based, social networking physical activity intervention for insufficiently active adults delivered via Facebook app: randomized controlled trial. J Med Internet Res.

[23] Quinn CC, Sareh PL, Shardell ML, Terrin ML, Barr EA, Gruber-Baldini AL (2014). Mobile diabetes intervention for glycemic control: impact on physician prescribing. J Diabetes Sci Technol.

[24] Krishnan A, Cravero C (2017). A multipronged evidence-based approach to implement mHealth for underserved HIV-infected populations. Mobile Media Commun.

[25] Heber E, Lehr D, Ebert DD, Berking M, Riper H (2016). Web-based and mobile stress management intervention for employees: a randomized controlled trial. J Med Internet Res.

[26] Cho JH, Lee HC, Lim DJ, Kwon HS, Yoon KH (2009). Mobile communication using a mobile phone with a glucometer for glucose control in Type 2 patients with diabetes: as effective as an Internet-based glucose monitoring system. J Telemed Telecare.

[27] Rosenstock IM (1974). Historical origins of the health belief model. Health Educ Monogr.

[28] Ajzen I (1991). The theory of planned behavior. Organ Behav Hum Dec.

[29] Bandura A (1986). Social Foundations of Thought and Action: A Social Cognitive Theory.

[30] Beck J (2011). Cognitive Behavior Therapy: Basics and Beyond.

[31] Noar SM, Benac CN, Harris MS (2007). Does tailoring matter? Meta-analytic review of tailored print health behavior change interventions. Psychol Bull.

[32] Snyder LB, Hamilton MA, Mitchell EW, Kiwanuka-Tondo J, Fleming-Milici F, Proctor D (2004). A meta-analysis of the effect of mediated health communication campaigns on behavior change in the United States. J Health Commun.

[33] Yang Q (2017). Are social networking sites making health behavior change interventions more effective? A meta-analytic review. J Health Commun.

[34] Oh B, Cho B, Han MK, Choi H, Lee MN, Kang H, Lee CH, Yun H, Kim Y (2015). The effectiveness of mobile phone-based care for weight control in metabolic syndrome patients: randomized controlled trial. JMIR Mhealth Uhealth.

[35] Patrick K, Raab F, Adams MA, Dillon L, Zabinski M, Rock CL, Griswold WG, Norman GJ (2009). A text message-based intervention for weight loss: randomized controlled trial. J Med Internet Res.

[36] Watts S, Mackenzie A, Thomas C, Griskaitis A, Mewton L, Williams A, Andrews G (2013). CBT for depression: a pilot RCT comparing mobile phone vs. computer. BMC Psychiatry.

[37] Newton J, Marker A, Allen H, Machtmes R, Han H, Johnson W, Schuna JM, Broyles ST, Tudor-Locke C, Church TS (2014). Parent-targeted mobile phone intervention to increase physical activity in sedentary children: randomized pilot trial. JMIR Mhealth Uhealth.

[38] Patrick K, Norman GJ, Davila EP, Calfas KJ, Raab F, Gottschalk M, Sallis JF, Godbole S, Covin JR (2013). Outcomes of a 12-month technology-based intervention to promote weight loss in adolescents at risk for type 2 diabetes. J Diabetes Sci Technol.

[39] de Niet J, Timman R, Bauer S, van den Akker E, Buijks H, de Klerk C, Kordy H, Passchier J (2012). The effect of a short message service maintenance treatment on body mass index and psychological well-being in overweight and obese children: a randomized controlled trial. Pediatr Obes.

[40] Irvine AB, Russell H, Manocchia M, Mino DE, Cox GT, Morgan R, Gau JM, Birney AJ, Ary DV (2015). Mobile-Web app to self-manage low back pain: randomized controlled trial. J Med Internet Res.

[41] Mira JJ, Navarro I, Botella F, Borrás F, Nuño-Solinís R, Orozco D, Iglesias-Alonso F, Pérez-Pérez P, Lorenzo S, Toro N (2014). A Spanish pillbox app for elderly patients taking multiple medications: randomized controlled trial. J Med Internet Res.

[42] Kerr DA, Harray AJ, Pollard CM, Dhaliwal SS, Delp EJ, Howat PA, Pickering MR, Ahmad Z, Meng X, Pratt IS, Wright JL, Kerr KR, Boushey CJ (2016). The connecting health and technology study: a 6-month randomized controlled trial to improve nutrition behaviours using a mobile food record and text messaging support in young adults. Int J Behav Nutr Phys Act.

[43] McGillicuddy JW, Gregoski MJ, Weiland AK, Rock RA, Brunner-Jackson BM, Patel SK, Thomas BS, Taber DJ, Chavin KD, Baliga PK, Treiber FA (2013). Mobile health medication adherence and blood pressure control in renal transplant recipients: a proof-of-concept randomized controlled trial. JMIR Res Protoc.

[44] Wayne N, Perez DF, Kaplan DM, Ritvo P (2015). ealth coaching reduces HbA1c in type 2 diabetic patients from a lower-socioeconomic status community: a randomized controlled trial. J Med Internet Res.

[45] Bhati S (2015). George Mason University.

[46] Rote AE, Klos LA, Brondino MJ, Harley AE, Swartz AM (2015). The efficacy of a walking intervention using social media to increase physical activity: a randomized trial. J Phys Act Health.

[47] Lee M, Wu H, Lin J, Tan T, Chan P, Chen Y (2014). Development and evaluation of an E-health system to care for patients with bladder pain syndrome/interstitial cystitis. Int J Urol.

[48] Lappalainen P, Kaipainen K, Lappalainen R, Hoffrén H, Myllymäki T, Kinnunen M, Mattila E, Happonen AP, Rusko H, Korhonen I (2013). Feasibility of a personal health technology-based psychological intervention for men with stress and mood problems: randomized controlled pilot trial. JMIR Res Protoc.

[49] Marcolino MS, Oliveira JA, D'Agostino M, Ribeiro AL, Alkmim MB, Novillo-Ortiz D (2018). The impact of mHealth interventions: systematic review of systematic reviews. JMIR Mhealth Uhealth.

[50] Hunter J, Schmidt F (2004). Methods of Meta-Analysis: Correcting error and bias in research findings.

[51] Rosenthal R (1991). Meta-Analytic Procedures for Social Research.

[52] Hedges LV, Olkin I (1985). Statistical Methods for Meta-Analysis.

[53] Sutton AJ, Cooper BS, Hedges LV, Valentine JC (2009). Publication bias. The Handbook of Research Synthesis and Meta-Analysi.

[54] Raudenbush SW, Cooper BS, Hedges LV, Valentine JC (2009). Analyzing effect sizes: random-effects models. The Handbook of Research Synthesis and Meta-Analysis.

[55] Borenstein M, Hedges LV, Higgins JP, Rothstein HR (2009). Introduction to Meta-Analysis.

[56] Schmidt FL, Hunter JE (1999). Comparison of three meta-analysis methods revisited: an analysis of Johnson, Mullen, and Salas (1995). J Appl Psychol.

[57] Choi J, Lee JH, Vittinghoff E, Fukuoka Y (2016). mHealth physical activity intervention: a randomized pilot study in physically inactive pregnant women. Matern Child Health J.

[58] Abraham AA, Chow WC, So HK, Yip BH, Li AM, Kumta SM, Woo J, Chan S, Lau EY, Nelson EAS (2015). Lifestyle intervention using an internet-based curriculum with cell phone reminders for obese Chinese teens: a randomized controlled study. PLoS One.

[59] Baron JS, Hirani S, Newman SP (2017). A randomised, controlled trial of the effects of a mobile telehealth intervention on clinical and patient-reported outcomes in people with poorly controlled diabetes. J Telemed Telecare.

[60] Chau JP, Lee DT, Yu DS, Chow AY, Yu W, Chair S, Lai AS, Chick Y (2012). A feasibility study to investigate the acceptability and potential effectiveness of a telecare service for older people with chronic obstructive pulmonary disease. Int J Med Inform.

[61] Chow CK, Redfern J, Hillis GS, Thakkar J, Santo K, Hackett ML, Jan S, Graves N, de Keizer L, Barry T, Bompoint S, Stepien S, Whittaker R, Rodgers A, Thiagalingam A (2015). Effect of lifestyle-focused text messaging on risk factor modification in patients with coronary heart disease: a randomized clinical trial. J Am Med Assoc.

[62] Cobb NK, Poirier J (2014). Effectiveness of a multimodal online well-being intervention: a randomized controlled trial. Am J Prev Med.

[63] Fountoulakis S, Papanastasiou L, Gryparis A, Markou A, Piaditis G (2015). Impact and duration effect of telemonitoring on ΗbA1c, BMI and cost in insulin-treated Diabetes Mellitus patients with inadequate glycemic control: a randomized controlled study. Hormones (Athens).

[64] Frederix I, Hansen D, Coninx K, Vandervoort P, Vandijck D, Hens N, Van Craenenbroeck E, Van Driessche N, Dendale P (2015). Medium-term effectiveness of a comprehensive internet-based and patient-specific telerehabilitation program with text messaging support for cardiac patients: randomized controlled trial. J Med Internet Res.

[65] Istepanian RS, Zitouni K, Harry D, Moutosammy N, Sungoor A, Tang B, Earle KA (2009). Evaluation of a mobile phone telemonitoring system for glycaemic control in patients with diabetes. J Telemed Telecare.

[66] Johnson KB, Patterson BL, Ho YX, Chen Q, Nian H, Davison CL, Slagle J, Mulvaney SA (2016). The feasibility of text reminders to improve medication adherence in adolescents with asthma. J Am Med Inform Assoc.

[67] Kamal AK, Shaikh Q, Pasha O, Azam I, Islam M, Memon AA, Rehman H, Akram MA, Affan M, Nazir S, Aziz S, Jan M, Andani A, Muqeet A, Ahmed B, Khoja S (2015). A randomized controlled behavioral intervention trial to improve medication adherence in adult stroke patients with prescription tailored Short Messaging Service (SMS)-SMS4Stroke study. BMC Neurol.

[68] Kirwan M, Vandelanotte C, Fenning A, Duncan MJ (2013). Diabetes self-management smartphone application for adults with type 1 diabetes: randomized controlled trial. J Med Internet Res.

[69] Laing BY, Mangione CM, Tseng C, Leng M, Vaisberg E, Mahida M, Bholat M, Glazier E, Morisky DE, Bell DS (2014). Effectiveness of a smartphone application for weight loss compared with usual care in overweight primary care patients: a randomized, controlled trial. Ann Intern Med.

[70] Nguyen B, Shrewsbury VA, O'Connor J, Steinbeck KS, Hill AJ, Shah S, Kohn MR, Torvaldsen S, Baur LA (2013). Two-year outcomes of an adjunctive telephone coaching and electronic contact intervention for adolescent weight-loss maintenance: the Loozit randomized controlled trial. Int J Obes (Lond).

[71] Quinn CC, Clough SS, Minor JM, Lender D, Okafor MC, Gruber-Baldini A (2008). WellDoc mobile diabetes management randomized controlled trial: change in clinical and behavioral outcomes and patient and physician satisfaction. Diabetes Technol Ther.

[72] Youl PH, Soyer HP, Baade PD, Marshall AL, Finch L, Janda M (2015). Can skin cancer prevention and early detection be improved via mobile phone text messaging? A randomised, attention control trial. Prev Med.

[73] Zairina E, Abramson MJ, McDonald CF, Li J, Dharmasiri T, Stewart K, Walker SP, Paul E, George J (2016). Telehealth to improve asthma control in pregnancy: a randomized controlled trial. Respirology.

[74] Allman-Farinelli M, Partridge SR, McGeechan K, Balestracci K, Hebden L, Wong A, Phongsavan P, Denney-Wilson E, Harris MF, Bauman A (2016). A mobile health lifestyle program for prevention of weight gain in young adults (TXT2BFiT): nine-month outcomes of a randomized controlled trial. JMIR Mhealth Uhealth.

[75] Hebden L, Cook A, van der Ploeg HP, King L, Bauman A, Allman-Farinelli M (2013). A mobile health intervention for weight management among young adults: a pilot randomised controlled trial. J Hum Nutr Diet.

[76] Ramachandran A, Snehalatha C, Ram J, Selvam S, Simon M, Nanditha A, Shetty AS, Godsland IF, Chaturvedi N, Majeed A, Oliver N, Toumazou C, Alberti KG, Johnston DG (2013). Effectiveness of mobile phone messaging in prevention of type 2 diabetes by lifestyle modification in men in India: a prospective, parallel-group, randomised controlled trial. Lancet Diabetes Endocrinol.

[77] Holmen H, Torbjørnsen A, Wahl AK, Jenum AK, Småstuen MC, Arsand E, Ribu L (2014). A mobile health intervention for self-management and lifestyle change for persons with type 2 diabetes, part 2: one-year results from the Norwegian randomized controlled trial RENEWING HEALTH. JMIR Mhealth Uhealth.

[78] Torbjørnsen A, Jenum AK, Småstuen M, Årsand E, Holmen H, Wahl A, Ribu L (2014). A low-intensity mobile health intervention with and without health counseling for persons with type 2 diabetes, part 1: Baseline and short-term results from a randomized controlled trial in the Norwegian part of RENEWING HEALTH. JMIR mHealth uHealth.

[79] Direito A, Jiang Y, Whittaker R, Maddison R (2015). Apps for IMproving FITness and increasing physical activity among young people: the AIMFIT pragmatic randomized controlled trial. J Med Internet Res.

[80] Arora S, Peters AL, Burner E, Lam CN, Menchine M (2014). Trial to examine text message-based mHealth in emergency department patients with diabetes (TExT-MED): a randomized controlled trial. Ann Emerg Med.

[81] Proudfoot J, Clarke J, Birch MR, Whitton AE, Parker G, Manicavasagar V, Harrison V, Christensen H, Hadzi-Pavlovic D (2013). Impact of a mobile phone and web program on symptom and functional outcomes for people with mild-to-moderate depression, anxiety and stress: a randomised controlled trial. BMC Psychiatry.

[82] Reid SC, Kauer SD, Hearps SJ, Crooke AH, Khor AS, Sanci LA, Patton GC (2011). A mobile phone application for the assessment and management of youth mental health problems in primary care: a randomised controlled trial. BMC Fam Pract.

[83] Constant D, de Tolly K, Harries J, Myer L (2014). Mobile phone messages to provide support to women during the home phase of medical abortion in South Africa: a randomised controlled trial. Contraception.

[84] Liguori S, Stacchini M, Ciofi D, Olivini N, Bisogni S, Festini F (2016). Effectiveness of an app for reducing preoperative anxiety in children: a randomized clinical trial. JAMA Pediatr.

[85] Hurling R, Catt M, Boni MD, Fairley BW, Hurst T, Murray P, Richardson A, Sodhi JS (2007). Using internet and mobile phone technology to deliver an automated physical activity program: randomized controlled trial. J Med Internet Res.

[86] Karhula T, Vuorinen AL, Rääpysjärvi K, Pakanen M, Itkonen P, Tepponen M, Junno U, Jokinen T, van Gils M, Lähteenmäki J, Kohtamäki K, Saranummi N (2015). Telemonitoring and mobile phone-based health coaching among finnish diabetic and heart disease patients: randomized controlled trial. J Med Internet Res.

[87] Bell AM, Fonda SJ, Walker MS, Schmidt V, Vigersky RA (2012). Mobile phone-based video messages for diabetes self-care support. J Diabetes Sci Technol.

[88] Bobrow K, Farmer AJ, Springer D, Shanyinde M, Yu LM, Brennan T, Rayner B, Namane M, Steyn K, Tarassenko L, Levitt N (2016). Mobile phone text messages to support treatment adherence in adults with high blood pressure (SMS-text adherence support [StAR]): a single-blind, randomized trial. Circulation.

[89] Cingi C, Yorgancioglu A, Cingi CC, Oguzulgen K, Muluk NB, Ulusoy S, Orhon N, Yumru C, Gokdag D, Karakaya G, Çelebi Ş, Çobanoglu HB, Unlu H, Aksoy MA (2015). The "physician on call patient engagement trial" (POPET): measuring the impact of a mobile patient engagement application on health outcomes and quality of life in allergic rhinitis and asthma patients. Int Forum Allergy Rhinol.

[90] DeVito Dabbs A, Song MK, Myers BA, Li R, Hawkins RP, Pilewski JM, Bermudez CA, Aubrecht J, Begey A, Connolly M, Alrawashdeh M, Dew MA (2016). A randomized controlled trial of a mobile health intervention to promote self-management after lung transplantation. Am J Transplant.

[91] Gamito P, Oliveira J, Lopes P, Brito R, Morais D, Silva D, Silva A, Rebelo S, Bastos M, Deus A (2014). Executive functioning in alcoholics following an mHealth cognitive stimulation program: randomized controlled trial. J Med Internet Res.

[92] Hsu WC, Lau KH, Huang R, Ghiloni S, Le H, Gilroy S, Abrahamson M, Moore J (2016). Utilization of a cloud-based diabetes management program for insulin initiation and titration enables collaborative decision making between healthcare providers and patients. Diabetes Technol Ther.

[93] Levy N, Moynihan V, Nilo A, Singer K, Bernik LS, Etiebet MA, Fang Y, Cho J, Natarajan S (2015). The mobile insulin titration intervention (MITI) for insulin adjustment in an urban, low-income population: randomized controlled trial. J Med Internet Res.

[94] Markowitz JT, Cousineau T, Franko DL, Schultz AT, Trant M, Rodgers R, Laffel LMB (2014). Text messaging intervention for teens and young adults with diabetes. J Diabetes Sci Technol.

[95] Petrella RJ, Stuckey MI, Shapiro S, Gill DP (2014). Mobile health, exercise and metabolic risk: a randomized controlled trial. BMC Public Health.

[96] Newton KH, Wiltshire EJ, Elley CR (2009). Pedometers and text messaging to increase physical activity: randomized controlled trial of adolescents with type 1 diabetes. Diabetes Care.

[97] Yoon KH, Kim HS (2008). A short message service by cellular phone in type 2 diabetic patients for 12 months. Diabetes Res Clin Pract.

[98] Kim CS, Park SY, Kang JG, Lee SJ, Ihm SH, Choi MG, Yoo HJ (2010). Insulin dose titration system in diabetes patients using a short messaging service automatically produced by a knowledge matrix. Diabetes Technol Ther.

[99] Liu CT, Yeh YT, Lee TI, Li YC (2005). Observations on online services for diabetes management. Diabetes Care.

[100] Office of Disease Prevention Health Promotion.

[101] Cohen J (1988). Statistical Power Analysis for the Behavioral Sciences.

[102] Higgins JP, Thompson SG, Deeks JJ, Altman DG (2003). Measuring inconsistency in meta-analyses. Br Med J.

[103] Finitsis DJ, Pellowski JA, Johnson BT (2014). Text message intervention designs to promote adherence to antiretroviral therapy (ART): a meta-analysis of randomized controlled trials. PLoS One.

[104] Liang X, Wang Q, Yang X, Cao J, Chen J, Mo X, Huang J, Wang L, Gu D (2011). Effect of mobile phone intervention for diabetes on glycaemic control: a meta-analysis. Diabet Med.

[105] Foster D, Linehan C, Kirman B, Lawson S, James G (2010). Motivating physical activity at work: using persuasive social media for competitive step counting.

[106] Kang Y, Cappella J, Fishbein M (2006). The attentional mechanism of message sensation value: interaction between message sensation value and argument quality on message effectiveness. Commun Monogr.

[107] Dutta-Bergman MJ (2004). Complementarity in consumption of news types across traditional and new media. J Broadcast Electron Media.

[108] Bandura A, Simon KM (1977). The role of proximal intentions in self-regulation of refractory behavior. Cogn Ther Res.

[109] Bull S, Ezeanochie N (2016). From Foucault to Freire through Facebook: toward an integrated theory of mHealth. Health Educ Behav.

[110] Brewin CR (1996). Theoretical foundations of cognitive-behavior therapy for anxiety and depression. Annu Rev Psychol.

[111] Petty RE, Cacioppo JT (1981). Attitudes and Persuasion: Classic and Contemporary Approaches.

[112] Lee H, Chae D, Cho S, Kim J, Yoo R (2016). Influence of a community-based stretching intervention on the health outcomes among Korean-Chinese female migrant workers in South Korea: A randomized prospective trial. Jpn J Nurs Sci.

