# Supplementary material for: The Comparative Effectiveness of Mobile Phone Interventions in Improving Health Outcomes: Meta-Analytic Review
Source: JMIR Mhealth Uhealth. 2019 Apr 3;7(4):e11244. doi: 10.2196/11244 (PMC6468337; doi:10.2196/11244)
Supplement: Multimedia Appendix 1 [file mhealth_v7i4e11244_app1.pdf]

Table 1. Summary of Studies Included in the Meta-Analysis.

	Study	ES	V	N	Age	Subgroup	Topic	Theory	Use Type	Outcome
1	Abraham (2015) [58]	0.58	0.10	32	14.4	Obesity clinic patients	Obesity - BMI, physical activity	HBM, TTM, SCT, CIPT	SMS	BMI
2	Allman-Farinelli (2016) [74]	0.38<br>0.42	0.02<br>0.02	248	27.6	Overweight/obese young adults	Obesity - nutrition, physical activity	TTM	SMS	Weight
3	Arora (2014) [80]	0.21	0.03	128	50.7	ED patients with diabetes	Diabetes management	HBM	SMS	HbA1c
4	Baron (2016) [59]	0.23	0.05	81	57.1	Patients with poorly controlled diabetes	Diabetes management	SCT, illness beliefs model, TAM	Telehealth	HbA1c
5	Bell (2012) [88]	0.00<br>0.05<br>0.24	0.06<br>0.06<br>0.06	64	58	Patients with poorly controlled diabetes	Diabetes management		SMS (video)	HbA1c
6	Bhati (2015) [45]	0.25	0.12	34	26.6	First-time mothers	Mental health & postpartum depression	HBM	SMS & app	Sleep disturbance
7	Block (2015) [8]	0.60<br>1.65	0.01<br>0.02	339	55	Pre-diabetic individuals	Diabetes risk - physical activity, healthy eating, weight loss	Learning theory, SCT, TPB	App	HbA1c<br>Fasting glucose
8	Bobrow (2016) [89]	0.09<br>0.13	0.00	915	54.3	Adult clinic patients with hypertension	Hypertension - systolic blood pressure	IBM	SMS	Systolic blood pressure
9	Chau (2012) [60]	-.47<br>0.38	0.19<br>0.10	40	72.9	COPD patients	Self-care management for older adults with COPD	SMT, SCT	Tracking oxygen saturation, pulse rate, and respiration rate	Hospital readmissions,<br>Pulmonary function
10	Cho (2009) [26]	0.27<br>-0.24<br>-0.17	0.06<br>0.06<br>0.06	69	48.2	Type 2 diabetes patients	Diabetes management		SMS & Telehealth	HbA1c<br>Mean fasting plasma glucose<br>2-hr postprandial glucose

11	Choi (2016) [57]	0.22	0.13	30	33.7	Pregnant women	Physical activity, depressive symptoms	SCT	App	Depressive symptoms
12	Chow (2015) [61]	0.15	0.01	710	57.6	CHD patients	CHD risk (including BMI) and health behaviors	Control theory, IMBSM, SCT, TPB, TRA	SMS	Cholesterol, BP, BMI, physical activity, diet, medications
13	Cingi (2015) [90]	0.59	0.03	139	31.1	Allergic rhinitis patients	Allergic rhinitis management & medication		App	QOL
		0.77	0.05	89	32.8	Asthma patients	Asthma management & medication			Asthma control
14	Cobb (2014) [62]	0.15	0.00	1502	42.5	Adults	Well-being	Game mechanics, SCT		Well-being
15	Constant (2014) [83]	0.26	0.01	381	25.8	Women undergoing abortion	Support for women undergoing abortion		SMS	Anxiety
		0.10	0.01							Socially negative emotions
		0.17	0.01							Internally negative emotions
16	De Niet (2012) [39]	0.05	0.03	141	9.9	Overweight/obese children	Lifestyle behavior and weight maintenance for overweight/obese children	Social learning theory, SCT, SRT	SMS	BMI
17	DeVito Dabbs (2016) [91]	0.18	0.04	201	62	Lung transplant recipients	Self-management after transplant	Orem's theory of self-care	App	Re-hospitalization
18	Direito (2015) [79]	0.45	0.12	34	15.7	Inactive healthy adults	Physical activity	SRT	App	Cardiorespiratory fitness
		0.36	0.13	32						
19	Du (2016) [7]	-.41	0.07	60	36	Adults	Physical activity and nutrition	TPB, SCT	App	Perceived stress
		-.62	0.07	64					SMS (video)	A1c
		0.20	0.07	60					App	Cardiorespiratory fitness
		0.00	0.06	64						
20	Fountoulakis (2015) [63]	1.01	0.05	105	55.3	Diabetes patients with inadequate glycemic control	Diabetes management	SMT, SCT	Telemonitoring	HbA1c
		0.68	0.05							

21	Frederix (2015) [64]	0.34	0.03	139	61	CVD patients	CVD	SMT, SCT	Telemonitoring & SMS	Peak aerobic capacity
22	Gamito (2014) [92]	-0.21	0.07	54	45.5	Patients with Alcohol Dependence Syndrome	Alcohol dependency		Apps for cognitive functioning	General cognitive abilities
23	Hebden (2014) [75]	0.12	0.08	51	22.8	Young adults	Physical activity, eating behaviors	TTM	App & SMS	BMI
24	Heber (2016) [25]	0.94	0.02	264	43.3	Employees with stress symptoms	Stress	Lazarus's transactional model of stress	SMS	Perceived stress
		1.10	0.02							
25	Holmen (2014) [77]	-.01	0.05	81	57	Diabetics with elevated HbA1C	Diabetes self-management	TTM	App	HbA1c
		0.13	0.05							
26	Hsu (2016) [93]	0.65	0.11	40	53.6	Patients with Type 2 diabetes	Diabetes medication management	Cognitive apprenticeship model, situated learning theory	App, in-app texts, & virtual visits	HbA1c
27	Hurling (2007) [85]	0.45	0.06	77	40.4	Healthy adults	Physical activity	Social comparison, decisional balance, ELM, goal theory	App	BMI
		0.49	0.06						SMS	Body fat
		0.01	0.05						App	Diastolic BP
		0.03	0.05							Systolic BP
28	Irvine (2015) [40]	0.21	0.01	398		Adults with back pain	Low back pain	SCT, TPB	App	Back pain
		0.23	0.01							
		0.21	0.01							
		0.23	0.01							
		0.10	0.01							Functionality, QOL, well-being
		0.28	0.01							
		0.11	0.01							
		0.30	0.01							
29	Istepanian (2009) [65]	0.24	0.02	137	58.6	Diabetes patients	Diabetes self-management	SMT, SCT	Telemonitoring	HbA1c
30	Johnson (2016) [66]	0.45	0.04	87	14.1	Adolescents	Medication adherence	SMT, SCT	App & SMS	QOL

31	Kamal (2015) [67]	0.27	0.02	200	56.9	Stroke survivors	Medication adherence	HBM, SCT	SMS	Diastolic BP
32	Karhula (2015) [87]	0.00<br>-.09<br>0.15<br>-.07	0.02<br>0.02<br>0.02<br>0.02	201<br>204<br>230	66.2<br><br>69.1	Heart disease patients<br><br>Diabetes patients	Health-related QOL for diabetic patients<br>Health-related QOL for heart disease patients	Wagner's chronic care model	App for Telemonitoring	Physical QOL<br>Mental QOL<br>Physical QOL<br>Mental QOL
33	Kerr (2016) [42]	0.12<br>0.39	0.02<br>0.02	147<br>141	24.6<br>24.3	Young adults	Diet/nutrition	SDT	App & Text<br>App	Body weight
34	Kim (2010) [99]	0.34	0.09	92		Patients with Type 2 diabetes	Diabetes management		SMS & Telehealth	HbA1c
35	Kirwan (2013) [68]	1.33<br>1.33	0.08<br>0.09	60<br>53	35.2	Patients with diabetes	Diabetes self-management	SMT, SCT	App & Text	HbA1c
36	Laing (2014) [69]	0.10<br>0.08	0.02<br>0.02	162<br>157	43.3	Adults with BMI > 25	Weight loss	SCT, SRT	MFP app	Weight loss
37	Lappalainen (2013) [48]	0.39<br>0.56	0.17<br>0.18	23	47.1	Males with stress, exhaustion, or sleeping problems	Stress-related problems		App	Psychological symptoms<br>Depression
38	Lee (2017) [86]	0.28<br>0.33<br>0.13<br>0.19<br>0.20<br>0.49<br>0.10<br>0.05	0.07<br>0.07<br>0.13<br>0.07<br>0.08<br>0.08<br>0.08<br>0.08	59	52.6	Female workers	Stretching	SCT, social support	SMS	Flexibility<br>Back strength<br>Flexibility<br>Back strength<br>Flexibility<br>Back strength<br>Flexibility<br>Back strength
39	Lee (2014) [47]	0.53<br>-.45<br>0.71<br>0.64<br>0.90<br>0.60<br>0.46<br>0.66	0.06<br>0.06<br>0.06<br>0.06<br>0.06<br>0.06<br>0.06<br>0.06	65	48	BPS/IC patients	Alleviate pain and increase QOL		SMS	Physical function<br>Role physical<br>Bodily pain<br>General health<br>Vitality<br>Social function<br>Role emotion<br>Mental health
40	Levy (2015) [94]	1.39	0.14	60	46.7	Patients with diabetes	Diabetes management	SMT, SCT	SMS	Reached optimal dose

41	Liguori (2016) [84]	0.91	0.11	39	8.7	Children with a planned surgery	Preoperative anxiety		App	Preoperative anxiety
42	Liu (2011) [100]	0.73	0.05	89	52.3	Patients with moderate to severe asthma	Asthma control		Phone software program	Respiratory function
43	Markowitz (2015) [95]	0.27	0.04	90	18.7	YYAs with diabetes	Diabetes management		SMS	PA and healthy food efficacy
44	McGillicuddy (2013) [43]	0.96<br>0.98	0.24<br>0.24	19	50.42	Recipients of a kidney transplant	Medical adherence, BP	SDT	SMS	Systolic BP<br>diastolic BP
45	Mira (2014) [41]	0.25<br>0.48	0.04<br>0.04	99	71.9	Elderly multi-morbid patients	Medical adherence		App (ALICE)	Medication error<br># of missed doses
46	Montes (2012) [16]	0.28<br>0.04	0.02<br>0.02	251	39.7	Adults with schizophrenia	Medical adherence		SMS	QOL
47	Newton (2014) [37]	0.20	0.15	27	8.7	Sedentary children	Physical activity	SCT	SMS	Sedentary time
48	Newton (2009) [97]	-0.09<br>0.03<br>0.43	0.05<br>0.05<br>0.05	78	14.4	Adolescents with Type 1 diabetes	Diabetes management		SMS & pedometer	A1c
49	Nguyen (2013) [70]	-.08<br>0.22	0.04<br>0.04	83	14.1	Overweight or obese youth	Weight loss	SCT	SMS	Body weight
50	Oh (2015) [34]	0.44<br>0.44	0.01<br>0.01	140	9.9	Obese patients	Weight control		SMS	Body weight
51	Patrick (2013) [38]	0.27<br>0.25<br>0.30<br>0.44	0.09<br>0.10<br>0.09<br>0.10	334<br><br>46	48.42<br><br>14.13	Adolescents at risk of diabetes	Weight control	Behavioral determinants model and TTM	SmartCare app<br><br>SMS	Body weight<br>BMI
52	Patrick (2009) [35]	0.62	0.08	46	44.9	Overweight adults	Weight loss	SMT, SCT	SMS	Body fat
53	Petrella (2014) [96]	-.30	0.03	127	56.74	Adults at metabolic risk	Diabetes management		App (Healthany-where)	Systolic BP
54	Proudfoot (2013) [81]	0.27<br>0.39<br>0.06	0.01<br>0.01<br>0.02	321<br>324<br>267	38.9	Patients with depression, anxiety or stress	Mental health		SMS	Depression
55	Quinn (2008) [71]	0.79	0.14	30	51.04	Patients with Type 2 diabetes	Diabetes management	SCT, behavioral theories	Tracking software	A1C

56	Ramanchandran (2013) [76]	0.29	0.01	537	46	Men with impaired glucose tolerance	Diabetes prevention (physical activity & diet)	TTM	SMS	Type 2 diabetes incidence
57	Reid (2011) [82]	0.11	0.05	83	18.05	YYAs with mental health issue	Mental health management		App	Depression
		0.09	0.05	86						
		0.09	0.05	78						Emotional self-awareness
		0.59	0.05	82						
58	Shaw (2013) [17]	0.28	0.07	60	53.3	Obese adults	Weight loss	Regulatory focus theory, TTM, HTT	SMS	Weight loss
		0.32	0.07							
		0.31	0.06							
		0.38	0.06							
59	Torbjornsen (2014) [78]	-.17	0.05	79	57	Patients with Type 2 Diabetes	Self-management	TTM	App	HbA1c levels
		0.13	0.05	83						Self-management
		0.02	0.05	79						HbA1c levels
		0.48	0.05	84						self-management
60	Watts (2013) [76]	-.04	0.17	25	41	Patients with depression	Treatment program for depression		App (Get Happy)	Psychological distress
		-.09	0.18	23						
		0.48	0.17	25						Major depressive disorder
		1.31	0.22	23						
61	Wayne (2015) [44]	0.45	0.04	97	53.2	Patients with Type 2 diabetes	Diabetes management		Connected Wellness Platform	HbA1c levels
		0.14	0.04							
62	Yoon (2008) [98]	1.14	0.09	51	47.2	Patients with Type 2 diabetes	Diabetes management		SMS	HbA1c
63	Youl (2015) [72]	0.65	0.01	330	32	Randomly selected adults	Skin cancer prevention and self-examination	SCT	SMS	Skin self-examination
		0.49	0.01	512						
64	Zarina (2016) [73]	0.27	0.06	69	31.4	Pregnant women with diabetes	Asthma control and QOL	SMT, SCT	App (Breathe-easy)	Asthma control
		0.35	0.06	67						QOL
		0.58	0.06	60			Alleviate pain and increase QOL			Asthma control
		0.79	0.06	67						QOL

Note. The study was named by the first author's last name and the year of publication. SMS = short messaging service. SCT = social cognition theory, IBM = integrated behavioral model, SMT= self-management theory, TPB = theory of planned behavior, TTM = transtheoretical model, HBM = health belief model, SDT= self-determination theory, SRT= self-regulation theory, CIPT = Consumer information processing theory, ELM = elaboration likelihood model, TAM = technology acceptance model, HTT = habituation-tedium theory, IMBSM = information-motivation-behavioral skills model, CVD = cardiovascular disease, QOL = QOL, BMI = body mass index, BP = blood pressure, YYA = youth and young adult.
